# The Prolonged Diagnostic Pathway of Young Adults (Aged 25–39) with Cancer in the United Kingdom: Results from the Young Adult Cancer Patient Journey Study

**DOI:** 10.3390/jcm10204646

**Published:** 2021-10-11

**Authors:** Victorien L. M. N. Soomers, Emma Lidington, Bhawna Sirohi, Michael A. Gonzalez, Anne-Sophie Darlington, Winette T. A. van der Graaf, Olga Husson

**Affiliations:** 1 Radboudumc, Department of Medical Oncology, 6525 GA Nijmegen, The Netherlands; 2 The Royal Marsden NHS Foundation Trust, London SW3 6JJ, UK; emma.lidington@rmh.nhs.uk (E.L.); w.vd.graaf@nki.nl (W.T.A.v.d.G.); 3 Max Institute of Cancer Care, Max Healthcare, New Delhi 110024, India; bhawna.sirohi@maxhealthcare.com; 4 Imperial College Healthcare NHS Trust, London SW7 2BX, UK; Michael.gonzalez@icr.ac.uk; 5 Department of Health Sciences, University of Southampton, Southampton SO17 1BJ, UK; a.darlington@soton.ac.uk; 6 Department of Medical Oncology, Netherlands Cancer Institute, 1066 CX Amsterdam, The Netherlands; 7 Department of Medical Oncology, Erasmus MC Cancer Institute, Erasmus University Medical Centre, 3015 GD Rotterdam, The Netherlands; 8 The Institute of Cancer Research, London SM2 5NG, UK

**Keywords:** diagnostic pathway, patient interval, healthcare interval, time to diagnosis

## Abstract

Purpose: Teenagers and young adults (TYAs; aged 13–24) experience prolonged intervals to cancer diagnosis. Insight into diagnostic intervals in young adults (YAs; aged 25–39) and subgroups at risk for long intervals is lacking. We investigated the diagnostic pathway of YA cancer patients, examined patient and tumor characteristics associated with its length, and compared the patient interval length of our sample with a TYA cohort. Methods: In this cross-sectional survey YAs diagnosed with cancer in the UK in the past five years completed a questionnaire describing their patient (time from first symptom to first doctor consultation) and healthcare interval (from first consultation until consultation with a cancer specialist), sociodemographic, and clinical characteristics. Associations between characteristics and interval length were examined and compared with previously published data in TYAs. Results: Among 341 YAs the patient interval lasted ≥2 weeks, ≥1 month, and ≥3 months in 60%, 42%, and 21%, respectively, compared to 48%, 27%, and 12% in the TYA group. The healthcare interval lasted ≥2 weeks, ≥1 month, and ≥3 months in 62%, 40%, and 17% of YA patients, respectively. YAs with melanoma or cervical cancer were most likely to experience long intervals, whereas YAs with breast cancer and leukemia were most likely to experience short intervals. Conclusions: Most YAs were not seen by a cancer specialist within 2 weeks of GP consultation. Interval lengths in YAs were associated with cancer diagnosis. Patient intervals were longer among YAs than among TYAs. Our study highlights long diagnostic pathways among YAs and calls for more awareness among healthcare professionals about malignancies in this age group.

## 1. Introduction

Cancer in adolescence and young adulthood (AYA), defined as patients aged 15–39 at cancer diagnosis, is uncommon, accounting for 5% of all cancer diagnoses [1]. Leukemia, lymphoma, testicular cancer, and thyroid cancer are the most common cancers among 15 to 24-year-olds, while breast cancer and melanoma are most common among 25–39-year-olds [2].

AYA cancer patients face unique developmental, physical, and psychosocial issues that make adjustment to their disease and health maintenance challenging [3]. AYAs describe unsatisfactory care experiences such as lack of recognition of their autonomy by healthcare providers (HCPs), lack of peer support, and inappropriate care environments [4,5]. To address these issues, the United Kingdom (UK) has rapidly expanded the availability of dedicated services for teenagers and young adults (TYA) ages 13 to 24. In contrast, no age-specific care services are available for young adult (YA) cancer patients aged 25 to 39 years.

Historically, progress in survival for AYAs has lagged behind both children and older adults, at least partly due to a prolonged diagnostic pathway [6,7,8]. Recently, we and others showed this gap in survival has closed for most, but not all tumors [9,10]. Early diagnosis of cancer is key to facilitate the start of treatment and can improve psychosocial and clinical outcomes [11,12,13]. The cause of prolonged diagnosis among AYA is likely to be multifactorial [14,15] and may include a lack of awareness amongst AYAs and HCPs, heterogenous and non-specific symptoms, and the rarity of cancer at this age. Reducing time to diagnosis is a key area for improving cancer care in the National Health Service [16]. The BRIGHTLIGHT study, assessing specialist care for TYAs with cancer in England [17], is the largest study among TYA patients looking at diagnostic timeliness [15]. In this study, over a quarter of participants (27%) waited more than one month to approach an HCP about symptoms [15].

Although age-specific guidelines to improve diagnostic timeliness in TYAs have been developed in the UK, for YAs, no specific guidance exists [18]. Information regarding YA’s diagnostic pathway is lacking and often obscured in studies of older adults where most patients are over age 50. As life events and the distribution of cancer types among YAs are distinct compared to older adults, available evidence cannot be extrapolated to YAs.

To improve healthcare services for YAs, we aim to describe the diagnostic pathway of patients aged 25–39 at diagnosis, identify factors associated with a prolonged pathway, compare the time from first symptom to doctor consultation in YAs with that in TYAs, and describe suggestions made by YAs to improve the diagnostic pathway.

## 2. Methods

### 2.1. Study Design and Participants

In this cross-sectional observational study, we invited all surviving patients diagnosed with cancer (ICD–10 codes C00–C97) aged 25–39 years treated at a participating trust (The Royal Marsden Hospital NHS Foundation Trust, East Suffolk and North Essex NHS Foundation Trust, University Hospital Southampton NHS Foundation Trust, Barts Health NHS Trust, Imperial College Healthcare NHS Trust, and East and North Hertfordshire NHS Trust). Patients were eligible if they were diagnosed in the last 5 years, able to communicate in English, and could complete questionnaires independently. Patients with a previous cancer diagnosis were excluded.

### 2.2. Ethical Approval

The Royal Marsden and Institute of Cancer Research Joint Committee on Clinical Research reviewed and sponsored the study (CCR4648). The Research Ethics Committee and Health Research Authority in the UK approved the study nationally (17/LO/0219).

### 2.3. Recruitment and Data Collection

Eligible patients received a letter from their treating physician explaining the purpose of the study. Patients provided informed consent before taking part. Data collection was conducted from May 2018 until March 2019 using PROFILES (www.profilesregistry.nl, accessed on 30 September 2021), a web-based system designed to collect patient-reported outcomes in cancer trials. Questionnaires could be completed online or upon request by pencil and paper.

## 3. Study Measures

Whilst the study was primarily designed to examine unmet supportive care needs of YAs, this paper describes secondary analyses to explore the diagnostic pathway of participants.

### 3.1. Demographic and Clinical Variables

The questionnaire package contained socio-demographic items, including age at diagnosis, gender, ethnicity, relationship status, educational level, and gross income per annum. Patients also self-reported clinical data including tumor type and comorbidities.

### 3.2. Diagnostic Pathway

**Figure 1:**
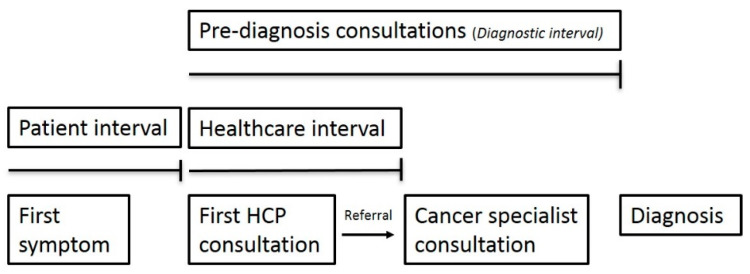
Diagnostic pathway.

The questionnaire package included a number of items about the diagnostic pathway, including items developed by the BRIGHTLIGHT group to assess the diagnostic pathway of TYAs [15,19]. We explored the patient and healthcare intervals and the number of pre-diagnosis consultations as a surrogate marker of diagnostic timeliness (Figure 1). The patient interval, as defined previously [20], encompasses the time between the first symptom and first consultation with an HCP. The healthcare interval is the time from the first HCP consultation until the first consultation with a cancer specialist. Interval items had categorical response options of under 1 week, 1–2 weeks, 2–4 weeks, 1–3 months, 3–6 months, 6–12 months, more than 12 months, or ‘I don’t know’. The number of pre-diagnosis consultations was measured with response options 0, 1, 2–3, or ‘4 times or more’.

An additional question assessed whether participants felt they were taken seriously by the first doctor they spoke to: “On a scale of 1 to 10, do you think your symptoms or concerns were taken seriously the first time you spoke to a doctor?”. A single free-text question asked for patient opinions on appropriate ways to reduce the time from symptom presentation to diagnosis.

### 3.3. Statistical Analysis

Descriptive statistics were reported for participants’ demographic and clinical data, patient and healthcare interval lengths, the number of consultations, and whether patients felt they were taken seriously. Mean and standard deviation are reported for continuous variables. Frequency and percentage are reported for categorical variables. For patient and healthcare intervals, we dichotomized interval lengths at three separate thresholds: <2 weeks versus ≥2 weeks, <1 month versus ≥1 month, and <3 months versus ≥3 months.

Available data from patients at the Royal Marsden Hospital NHS Foundation Trust was used for a non-responder analysis. Age at diagnosis, current age, cancer type, and years from diagnosis were captured for non-responders. The characteristics of responders and non-responders were compared using independent samples *t*-tests for continuous data and chi-square tests for categorical data.

We performed univariate logistic regression analyses to detect associations between categorical independent variables and the length of the patient and healthcare intervals dichotomized at 1 month following previous studies [15,21]. Odds ratios (OR) and 95% confidence intervals (95% CI) are presented. Independent samples *t*-tests were performed for continuous variables. We did not perform multivariable analysis because there were too few observations in each cancer type.

The number of pre-referral consultations is an indicator of diagnostic timeliness as patients experiencing more pre-referral consultations have longer intervals from symptom presentation to diagnosis [22]. We argue that two consultations are usually needed before referral, thus ≥4 consultations best reflect a prolonged interval. Therefore, we dichotomized diagnostic timeliness into <4 or ≥4 consultations. Fisher’s exact tests were performed to test associations between categorical variables and the number of consultations before diagnosis.

To compare our results with TYA patient intervals, we used data published by the BRIGHTLIGHT study group [15]. We were unable to compare the healthcare interval or number of consultations, as definitions and cut-off points between the two cohorts differed. We grouped carcinomas and combined all germ-cell tumors to make direct comparisons with the BRIGHTLIGHT cohort. Groups with too few observations or not occurring in both cohorts were excluded from the analysis. We reported frequency and percentage of patient intervals in both groups and tested the differences using Χ^2^ tests. As we had no access to the raw data from the BRIGHTLIGHT study, tests were limited to univariate analysis. Associations between patient characteristics and age group were restricted to single levels of the patient. If the expected number within a cell was smaller than five, Fisher’s exact tests were performed.

All missing data were assumed to be missing at random and only complete cases were analyzed. All statistical analyses were performed using IBM SPSS 25.0 (IBM, Armonk, NY, USA). Two-sided *p*-values of <0.05 were considered statistically significant.

### 3.4. Qualitative Analysis

We analyzed free-text responses using inductive coding followed by axial coding to group participants’ answers [23]. Two investigators independently coded the data (VS and OH). We describe the number of times each recommendation occurred.

## 4. Results

### 4.1. Participants

Of the 1657 invited patients, 348 completed the questionnaire (response rate 21%); 341 participants had complete healthcare interval data and were included in the analysis. The mean age was 33.3 years, 108 (32%) were male, and 288 (84%) were white (Table 1). Breast cancer and testicular cancer were the most common diagnoses. The mean time between diagnosis and questionnaire completion was 2.9 years (standard deviation 1.7).

### 4.2. Non-Responder Analysis

Responders and non-responders did not differ in age at diagnosis, current age, years from diagnosis, or cancer type (Appendix A).

### 4.3. Patient Interval

Patient interval data was completed by 307 participants. Seventy-eight percent first told a doctor about their symptoms, mostly their general practitioner (GP) (84%). A minority of patients were admitted as an emergency (4%) or were detected through screening (6%). Those detected through screening had breast (*n* = 2) or cervical cancer (*n* = 16). Half the participants with cervical cancer (*n* = 16) were not detected through screening. The majority (68%) of patients felt they were taken seriously by the first doctor they spoke to.

**Figure 2:**
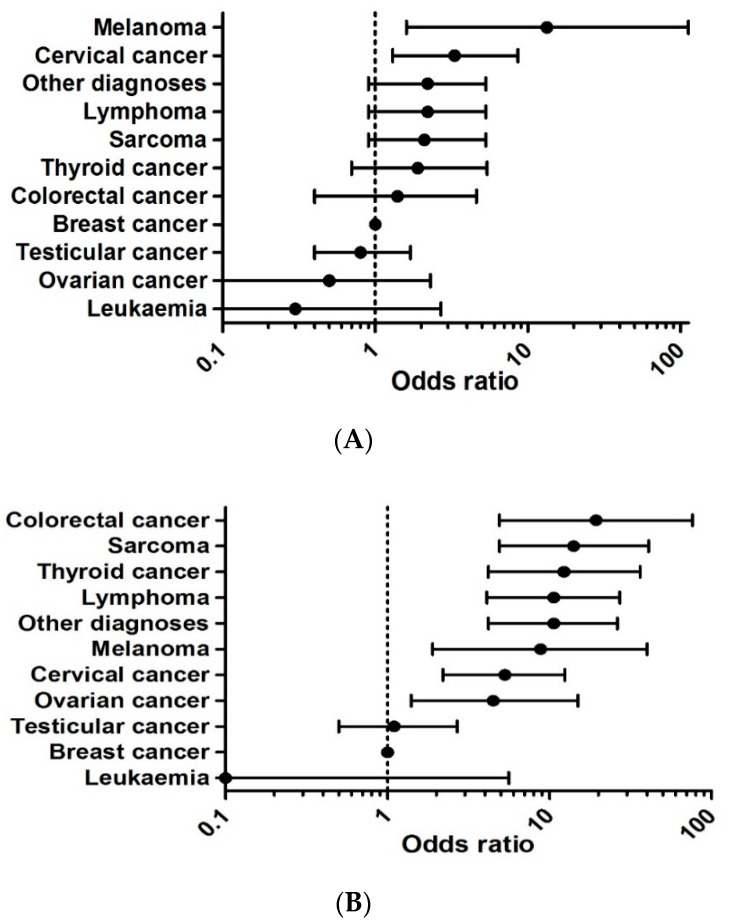

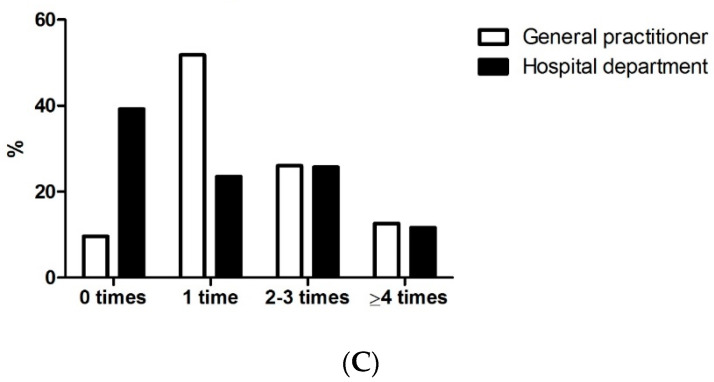
(**A**) Odds ratios of patient interval ≥1 month by diagnosis. (**B**) Odds ratios of healthcare interval ≥1 month by diagnosis. (**C**) Number of pre-diagnosis consultations.

Although 94% of participants experienced symptoms, the majority (60%) waited longer than two weeks before consulting a doctor. In 42% and 21% of cases, participants waited longer than one and three months, respectively (Table 1). Reasons for delaying included waiting to see whether symptoms would disappear spontaneously, thinking there was no need to go to the doctor, being too busy, and not wanting to bother the doctor unnecessarily. Patients with melanoma and cervical cancer had significantly higher odds of experiencing a patient interval greater than one month compared to those with breast cancer (Figure 2A). Gender, age, and ethnicity were not associated with patient interval length (Table 2).

### 4.4. Healthcare Interval

Most patients (62%) had a healthcare interval ≥2 weeks. Forty percent of patient intervals were ≥1 month and 17% ≥3 months (Table 1). Compared to breast cancer, all other cancer types except for leukemia and testicular cancer had significantly higher odds of experiencing a healthcare interval ≥1 month (Figure 2B). Gender, ethnicity, and the presence of a symptom were not associated with healthcare interval length. Patients with an interval ≥1 month were significantly younger than patients with an interval <1 month (Table 2).

Before receiving a diagnosis, 90% of patients spoke to their GP, 14% to an A&E doctor, 61% to a hospital doctor not in A&E, 9% to a walk-in center clinician, 2% to a polyclinic doctor, and 12% to another doctor. A considerable number of participants (13%) spoke to their GP or a hospital doctor other than in A&E (12%) ≥4 times before diagnosis (Figure 2C).

The number of consultations, regardless of location, was not associated with age, gender, or symptom presence (Table 3). Cancer type was associated with >4 GP consultations and >4 hospital doctor consultations. Participants diagnosed with leukemia, sarcoma, ovarian cancer, thyroid cancer, colorectal cancer, and “other diagnoses” most often had >4 GP consultations. Participants diagnosed with leukemia, lymphoma, sarcoma, testicular cancer, ovarian cancer, and “other diagnoses” most often had >4 hospital doctor consultations.

### 4.5. Comparison of Findings with TYA Population

The BRIGHTLIGHT cohort included 830 TYAs aged 12–24 at primary cancer diagnosis [15]. Their median age was 20 years, 55% were male, and 88% were white. Participants were diagnosed with lymphoma (32%), germ-cell tumors (19%), leukemia (13%), non-skin carcinomas (12%), bone cancer (10%), soft tissue sarcomas (6%), central nervous system neoplasms (4%), melanoma and skin carcinoma (4%), and unspecified (1%) (Table 4).

**Figure 3:**
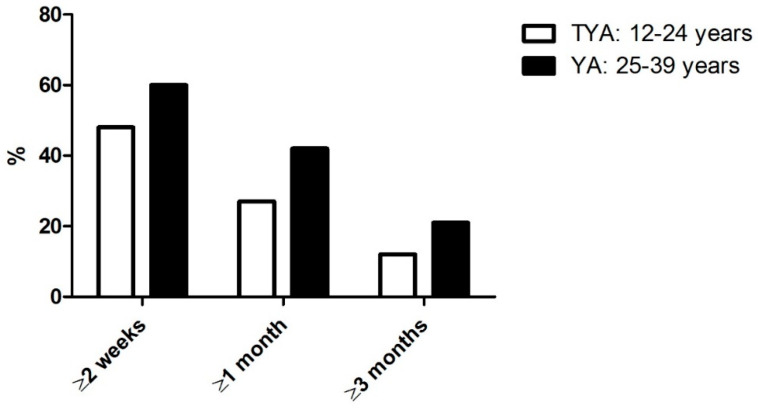
Proportion of participants by patient and interval length.

Complete patient interval data were reported for 748 TYAs. Compared to 341 YA participants, 48% versus 60% had a patient interval ≥2 weeks, 27% versus 42% ≥1 month, and 12% versus 21% ≥3 months, for TYA versus YA patients, respectively (Figure 3).

Among males, white respondents, and patients with lymphoma, YAs were significantly more likely to have a patient interval ≥1 month than TYA participants (Table 5). YAs were also significantly more likely to have a >2-week patient interval compared to TYAs among males and white patients, though this association was not significant among cancer diagnosis groups (Appendix A). When dichotomized at three months, YAs were significantly more likely to have a longer patient interval than TYA participants among males, white patients, or those diagnosed with lymphoma or sarcoma (Appendix A).

### 4.6. Suggestions for Improving the Diagnostic Pathway

Many patients (39%) gave a total of 191 suggestions to improve the diagnostic pathway. Themes included raising awareness of cancer in YAs and taking young people seriously, communication, and reducing passive waiting times. Table 6 shows exemplary quotes.

The majority (39%) of recommendations were about raising awareness among HCPs and YAs that age should not preclude cancer and taking YAs seriously (Table 3). Nearly a quarter (21%) suggested better communication, such as providing more information about investigations, not skirting around cancer suspicions, and not giving false reassurance. One in six (16%) thought the healthcare interval length could be reduced by shortening wait times for examinations, referrals and appointments, and sharing more information between institutions and departments.

A small number of remarks were about the patient interval, recommending that YAs should not wait to contact their GP with abnormalities and be persistent about getting a diagnosis (9%).

There were no major differences between groups, but participants with a healthcare interval ≥1 month more often remarked about raising awareness and being taken seriously (57%) and reducing waiting times for examinations, referrals, and appointments (50%).

## 5. Discussion

In this study, we investigated the diagnostic pathway of YA cancer patients, examined patient and tumor characteristics associated with the length of the diagnostic pathway, compared the patient interval length of our sample with a TYA cohort, and reported patients’ suggestions for improving the diagnostic pathway.

Both patient and healthcare intervals were long among a substantial proportion of participants. Forty-two percent of participants had patient intervals ≥1 month and 21% ≥3 months. Healthcare intervals were ≥1 month for 40% and ≥3 months for 17% of participants. Gender and ethnicity were not associated with diagnostic intervals or the number of consultations before diagnosis. Age was only associated with the healthcare interval, where age was slightly lower among patients with a >1 month interval. Remarkably, symptom presence at diagnosis did not influence healthcare interval length nor the number of GP or hospital doctor consultations.

Subtype-specific cancer diagnosis was associated with both patient and healthcare interval length and number of pre-diagnosis consultations. YAs with melanoma were most likely to wait ≥1 month before consulting a doctor but never had ≥4 hospital doctor consultations, as expected with identifiable presenting symptoms (an itching or bleeding pigmented lesion) of this cancer. The finding that identifiable presenting symptoms may lead to a short patient interval is supported by a sub-analysis of the BRIGHTLIGHT cohort, which shows 38% of participants with mole changes had a patient interval > 1 month [24].

YAs with cervical cancer were more likely to wait ≥1 month as well, and some had ≥4 GP consultations. Notably, half of these patients were not detected through screening. However, in the NHS one in four women skip cervical screening, with the proportion increasing to one in three among those aged 25 to 29 [25]. Unfortunately, our study did not ask cervical cancer patients not detected through screening whether they participated in the screening program. We therefore cannot conclude whether these were interval carcinomas occurring between two screening dates.

In breast cancer, one might expect a short patient interval as breast cancer patients form a distinct group compared to other cancer patients, given the general knowledge about the disease and its symptoms in the population. However, a third waited more than one month before consulting a doctor. We hypothesize this may be due to YAs having busy lives and not recognizing symptoms as caused by malignancy. Two participants with breast cancer reported being diagnosed through screening, possibly in a screening program for a hereditary cancer syndrome. The standard NHS screening program for breast cancer starts at age 50. Regarding the breast cancer healthcare interval, it is unsurprising that few participants had >4 GP (5%) or hospital (3%) consultations.

The NICE two-week-wait rule (TWW) states patients with a suspicion of cancer should be referred to a specialist in two weeks and additional investigations, including biopsies, should be carried out on one day [26]. Therefore, one would expect the healthcare interval to be shorter than two weeks for most participants. However, the healthcare interval lasted ≥2 weeks in 43% of YAs, and ≥1 month in 16%. As expected, few had a healthcare interval ≥3 months (2%). It is known that younger patients present less often via the TWW, and more often via non-TWW referrals or in emergency presentations, however, this may not be directly correlated with the healthcare interval, as the majority of patients will be diagnosed through emergency presentation [27].

Participants with diagnoses other than breast cancer were more likely to experience a healthcare interval ≥1 month. The only exception was leukemia, though these patients had many pre-diagnosis GP and hospital consultations. The need to perform additional investigations in leukemia patients to confirm the diagnosis may explain the high number, but most of these investigations can be undertaken and interpreted relatively quickly. Alternatively, patients with leukemia often present as an emergency, although this percentage is higher in TYAs than Yas [27].

Comparison with existing literature is difficult, as studies focusing solely on YAs 25–39 years of age are rare. This study enabled a direct comparison of YA and TYA patient intervals with findings from the BRIGHTLIGHT study. This showed that YAs in our study, in general, had longer patient intervals. Age-related factors may contribute to this difference, such as differing life priorities (e.g., having a job, taking care of children). The distribution of diagnoses may play an important role as well: the proportion of participants diagnosed with leukemia and lymphoma was larger in the TYA group, whereas carcinomas were diagnosed more often in the YA group. Participants who were male or white were more at risk of a longer patient interval when aged 25–39, compared to those aged 12–24. Furthermore, those diagnosed with lymphoma with a patient interval ≥1 month, or ≥3 months, were also more likely to be older. This was also true for patients with soft tissue sarcoma who had a patient interval ≥3 months. These findings are relevant and call for actions to increase awareness among YAs to reduce the patient interval.

Our findings support those of a European study, showing diagnostic routes among those aged 15–29 vary substantially, and an American study with patients aged 15–29 that found cancer diagnosis was significantly associated with interval length, whereas ethnicity, age, and gender were not [28]. Similarly, a National Cancer Intelligence Network report found that cancer diagnosis played a major role in determining how TYAs were likely to be referred [27].

A Danish study amongst AYAs (aged 15–39) reported GP consultations increased several months before cancer diagnosis, possibly reflecting low awareness of patients and HCPs that symptoms may be due to malignancy [29].

Although 68% of participants felt they were taken seriously in their first consultation, most suggestions to improve the diagnostic pathway were about taking YAs seriously, and not rejecting cancer as a possibility due to age. Additional recommendations were made about communication, and reducing passive waiting time, e.g., for additional examinations, referrals, or requesting information from other institutions. There were no major differences by interval length and most recommendations were not age specific.

To our knowledge, this is the first study to examine the diagnostic pathway of YA cancer patients, with various cancer diagnoses. However, this study has several limitations. First, intervals and the number of consultations were self-reported, potentially introducing recall bias. A generally consistent finding is that as the recall time increases, the ability to recall events degrades [30]. However, significant events, such as a cancer diagnosis, are less likely to be forgotten [30]. Furthermore, estimating the duration of an event is extremely stable [31,32]. To minimize the effect of recall bias, patients were asked to report the duration of intervals instead of dates, and questions were anchored to a life event (the cancer diagnosis).

Second, the study may be subject to selection bias as only 21% of invited participants responded, which is not unusual for studies among young adults with cancer. Data for the non-responder analysis was unfortunately only available from a selection of patients. However, this analysis does not show any differences in terms of age, time since diagnosis, or diagnosis.

Another cause of selection bias is the survivorship population in which we conducted our study. Not only will these people have had different tumor characteristics (e.g., lower stage at diagnosis), but they may also have had a different diagnostic pathway. Our results should thus be interpreted with this in mind.

Third, the distribution of tumors does not accurately reflect the incidence of cancers in YAs in the population [10]. For males, the most common cancers among YAs in the UK are testicular cancer, melanoma, and gastrointestinal tumors. For females, these are breast cancer, melanoma, and tumors of the genitourinary tract. Lymphoma and sarcoma are therefore overrepresented in our study, whilst melanoma and gastro-intestinal tumor may be underrepresented. We invited patients from hospitals in the South East, East, and London regions, who may have relatively more TWW referrals than those diagnosed in the North East [27]. Interval length may be underestimated when compared to the whole of England. Lastly, as subgroups were small, we were unable to perform adjusted analyses and the results should therefore be interpreted with caution.

Our findings highlight that cancer is still seen as a disease of the elderly. We recommend increasing awareness and gain better insight in the diagnostic pathway of patients aged 25–39 and raise awareness in the general public and among health care professionals to shorten time to diagnoses. Further research with a larger population is needed to confirm our findings with respect to identified risk groups, and to study the impact of a prolonged diagnostic pathway on clinical and patient-reported outcomes for YAs.

## 6. Conclusions

Patient and healthcare interval length is long in a substantial proportion of YA cancer patients. Diagnostic intervals were associated with cancer diagnosis, with YAs with melanoma or cervical cancer experiencing a long time to diagnosis, and YAs with breast cancer and leukemia experiencing a short diagnostic pathway. Compared to the TYA population, YA patients who were male, white, or diagnosed with lymphoma or STS, were more likely to experience a prolonged patient interval. Participants recommended improving the diagnostic pathway by raising awareness, enhancing communication, and reducing passive waiting time.

Take home message

The diagnostic pathway of YAs should be studied further and awareness about cancer in this age group should be increased. Healthcare providers should be aware of cancer incidence among young adults and provide adequate information and support for this age group.

## Figures and Tables

**Table 1:** Participant characteristics at time of survey.

	All Participants (*n* = 341)
	Mean (SD)
Age at Diagnosis in Years, Mean (SD)	33.3 (4.3)
	*n* (%)
Gender	
Male	108 (32)
Female	233 (68)
Ethnic group	
White	288 (84)
Non-White	53 (16)
Cancer diagnosis	
Breast cancer	113 (33)
Leukemia	9 (3)
Lymphoma	27 (8)
Sarcoma	22 (7)
Testicular cancer	52 (15)
Ovarian cancer	13 (4)
Melanoma	8 (2)
Thyroid cancer	20 (6)
Colorectal cancer	14 (4)
Cervical cancer	32 (9)
Other	30 (9)
Missing	1 (0)
Patient interval length	
(*n* = 307; non-exclusive)	
>2 weeks	185 (60)
>1 month	129 (42)
>3 months	63 (21)
Healthcare interval length	
(*n* = 341; non-exclusive)	
>2 weeks	210 (62)
>1 month	135 (40)
>3 months	59 (17)
Presence of symptom upon presentation	
Symptomatic	320 (94)
Asymptomatic	21 (6)
Relationship status	
Single	58 (17)
In a relationship	83 (24)
Married/civil partnership	189 (55)
Divorced	11 (3)
Educational level	
No education or primary school	2 (1)
Secondary school	32 (9)
Vocational	14 (4)
College	66 (19)
University	201 (59)
Other	26 (8)
gross income per annum	
GBP < 20,000	88 (26)
GBP 20,000–30,000	51 (15)
GBP > 30,000	162 (48)
Missing	40 (12)
Comorbidities	
0	177 (52)
1	114 (33)
≥2	50 (15)

**Table 2:** Participant characteristics by interval length.

	Patient Interval (*n* = 307)			Healthcare Interval (*n* = 341)		
	<1 Month	≥1 Month		<1 Month	>1 Month	
	Mean (SD)	Mean (SD)	*p*-Value #	Mean (SD)	Mean (SD)	*p*-Value #
Age at diagnosis in years	33.5 (4.3)	33.2 (4.4)	0.6	33.7 (4.2)	32.7 (4.3)	0.03
	*N* (%)	*N* (%)	OR (95% CI)	*N* (%)	*N* (%)	OR (95% CI)
All participants	178 (58)	129 (42)	NA	206 (60)	135 (40)	
Gender						
Male	57 (55)	46 (45)	1 (ref)	68 (63)	40 (37)	1 (ref)
Female	121 (59)	83 (41)	0.9 (0.5–1.4)	138 (59)	95 (41)	1.2 (0.7–1.9)
Ethnic group						
White	149 (57)	111 (43)	1 (ref)	172 (60)	116 (40)	1 (ref)
Non-White	29 (62)	18 (38)	0.8 (0.4–1.6)	34 (64)	19 (36)	0.8 (0.5–1.5)
Cancer diagnosis						
Breast cancer	72 (66)	38 (34)	1 (ref)	95 (84)	18 (16)	1 (ref)
Leukemia	6 (86)	1 (14)	0.3 (0.0–2.7)	8 (89)	1 (11)	0.1 (0.1–5.6)
Lymphoma	12 (46)	14 (54)	2.2 (0.9–5.3)	9 (33)	18 (67)	10.6 (4.1–27.2) ^
Sarcoma	9 (47)	10 (53)	2.1 (0.9–5.3)	6 (27)	16 (73)	14.1 (4.9–40.8) ^
Testicular cancer	35 (70)	15 (30)	0.8 (0.41–7)	43 (83)	9 (17)	1.1 (0.5–2.7)
Ovarian cancer	8 (80)	2 (20)	0.5 (0.12–3)	7 (54)	6 (46)	4.5 (1.4–15.0) ^
Melanoma	1 (12)	7 (88)	13.3 (1.6–111.8) ^	3 (38)	5 (63)	8.8 (1.9–40.1) ^
Thyroid cancer	8 (50)	8 (50)	1.9 (0.7–5.4)	6 (30)	14 (70)	12.3 (4.2–36.3) ^
Colorectal cancer	7 (58)	5 (42)	1.4 (0.4–4.6)	3 (21)	11 (79)	19.4 (4.9–76.3) ^
Cervical cancer	8 (36)	14 (64)	3.3 (1.3–8.6) ^	16 (50)	16 (50)	5.3 (2.2–12.4) ^
Other	12 (46)	12 (46)	2.2 (0.9–5.3)	10 (33)	20 (67)	10.6 (4.2–26.3) ^
Presence of symptom upon presentation						
Symptomatic	178 (58)	129 (42)	NA	190 (59)	130 (41)	1 (ref)
Asymptomatic	NA	NA		16 (76)	5 (24)	0.5 (0.1–1.3)

# Independent samples *t*-test; NA = not applicable; ^ *p* < 0.0; OR = odds ratio; CI = confidence interval. Ref = reference category.

**Table 3:** Participant characteristics with 4 or more pre-diagnosis consultations.

	≥4 GP Consultations		≥4 Hospital Consultations	
	Mean (SD)		Mean (SD)	
Age at diagnosis in years	32.3 (4.5) #		33.0 (4.3) #	
	*n* (%) ~	*p*-Value *	*n* (%) ~	*p*-Value *
All participants	42 (13)		37 (12)	
Gender		0.593		0.349
Male	11 (11)		14 (14)	
Female	31 (14)		23 (11)	
Ethnic group		1		1
White	36 (13)		31 (12)	
Non-White	6 (12)		6 (12)	
Cancer diagnosis		0.006 **		0.000 **
Breast cancer	5 (5)		3 (3)	
Leukemia	2 (25)		4 (50)	
Lymphoma	4 (15)		7 (27)	
Sarcoma	5 (23)		3 (14)	
Testicular cancer	2 (4)		6 (12)	
Ovarian cancer	3 (23)		2 (15)	
Melanoma	1 (13)		0 (0)	
Thyroid cancer	5 (25)		2 (11)	
Colorectal cancer	4 (29)		1 (7)	
Cervical cancer	4 (13)		2 (7)	
Other	7 (25)		7 (29)	
Presence of symptoms at presentation, *n* (%)		1		0.706
Symptomatic	40 (13)		36 (12)	
Asymptomatic	2 (11)		1 (6)	

~ Percentages do not add up to 100% as data per column is arranged as proportion of patients with certain characteristics within a certain time interval. * Fisher’s exact test; ** X^2^ test. # Independent samples *t*-test showed no differences between age and number of consultations.

**Table 4:** Characteristics of TYA and YA population.

	TYA	YA
	12–24 Years	25–39 Years
	*n* (%)	*n* (%)
All participants	748 (100)	307 (100)
Gender		
Male	419 (56)	103 (34)
Female	329 (44)	204 (66)
Ethnic group		
White	657 (88)	260 (85)
Non-White	91 (12)	47 (15)
Cancer diagnosis		
Leukemia	89 (12)	7 (2)
Lymphoma	248 (33)	26 (9)
Soft tissue sarcoma	41 (5)	19 (6)
Germ cell tumors	147 (20)	52 (17)
Melanoma	28 (4)	8 (3)
Carcinomas	87 (12)	152 (50)

TYA = teenagers and young adults; YA = young adults.

**Table 5:** Comparison of patient interval of TYA population with YA population.

	TYA (*n* = 748)		YA (*n* = 307)		TYA vs. YA
	<1 Month	>1 Month	<1 Month	>1 Month	>1 Month
	*n* (%)	*n* (%)	*n* (%)	*n* (%)	X^2^ *p*-Value
All participants	544 (73)	204 (27)	178 (58)	129 (42)	-
Gender					
Male	641 (74)	107 (26)	57 (55)	46 (45)	0
Female	651 (71)	97 (29)	121 (59)	83 (41)	0.12
Ethnic group					
White	566 (72)	182 (28)	149 (57)	111 (43)	0
Non-White	726 (76)	22 (24)	29 (62)	18 (38)	0.21
Cancer diagnosis					
Leukemia	726 (75)	22 (25)	6 (86)	1 (14)	0.36
Lymphoma	682 (73)	66 (27)	12 (46)	14 (54)	0.01
Soft tissue sarcoma	735 (68)	13 (32)	9 (47)	10 (53)	0.28
Germ cell tumors	712 (76)	36 (24)	37 (71)	15 (29)	0.69
Melanoma	734 (50)	14 (50)	1 (13)	7 (88)	0.06
Carcinomas	720 (68)	28 (32)	93 (61)	59 (39)	0.66

TYA = teenagers and young adults; YA = young adults.

**Table 6:** Quotes supporting qualitative analyses.

Raising awareness and taking young people seriously	“I didn’t come across many well-informed doctors before I was admitted to the ***. I think cancer was dismissed as a possible reason because I was relatively young and otherwise fit and healthy. No one took my tumor markers despite me having lumps/swelling. Perhaps my only suggestion is raising awareness with all doctors that age is not a reason to discount cancer if they can’t immediately identify the cause of a symptom. A blood test may have cut down my wait significantly.”
	“I rarely felt like I was being listened to and taken seriously as an individual who knew their own body. The GP only took me seriously when I found that a pre-existing lump in my breast had grown almost overnight, by which time it was too late. My sense was that the emergency/rapid response care was very good; but the preventative care and taking a holistic look at my symptoms in the early stages was completely overlooked.”
Communication	“I didn’t realize they could tell you on the day that its cancerous, I thought you had to wait for the results, so I was very unprepared and alone (without my husband/parent).”
	“My consultant sent me for a fine needle aspiration but told me this was fairly routine. I was not told this was a test for cancer. I feel that I should have been given at least some mild warning of the possibility of cancer by the consultant.”
Reducing passive waiting times	“Reducing the wait between being referred to seeing a specialist or having tests. It’s a very stressful and scary time.”
	“Share test results/scan info between trusts so tests do not have to repeated.”

## Data Availability

Data is contained within the article.

## Appendix A. Responders versus Non-Responders Royal Marsden Hospital

	**Responders (*N* = 209)**	**Non-Responders (*N* = 690)**
	Mean (SD)	Mean (SD)
Age at diagnosis in years	33.17 (4.41)	32.77 (4.32)
Age at time of study	35.82 (4.63)	35.42 (4.52)
Years from diagnosis	3.79 (1.78)	3.23 (1.77)
Cancer diagnosis		
Breast cancer	56	151
Leukemia	5	16
Lymphoma	9	44
Sarcoma	27	92
Testicular cancer	31	134
Ovarian cancer	7	10
Melanoma	11	25
Thyroid cancer	23	90
Colorectal cancer	10	35
Other	11	24
Missing	19	69
